# Aerodynamic Testing of a 3D-Printed Aircraft Model with a Post-Processed Surface

**DOI:** 10.3390/ma18173996

**Published:** 2025-08-26

**Authors:** Lucjan Setlak, Rafał Kowalik, Tomasz Lusiak

**Affiliations:** 1 Department of Avionics and Control Systems, Polish Air Force University, 08-521 Deblin, Poland; l.setlak@law.mil.pl; 2 Department of Thermodynamics, Lublin University of Technology, 20-618 Lublin, Poland; t.lusiak@polub.pl

**Keywords:** material structure, composite materials, aerodynamic properties, military supersonic aircraft

## Abstract

The subject of the research in this article were experimental tests of the M-346 Master aircraft model, carried out in a wind tunnel using the 3D printing method (FDM) in terms of the impact of surface post-processing technology on its aerodynamic characteristics. The measurements of key aerodynamic parameters concerned forces and moments in various airflow conditions taking into account variable angles of attack at a constant sideslip angle. The main purpose of the work was to verify the hypothesis that properly performed surface treatment significantly affects the accuracy of actual aerodynamic measurements in terms of solving the research problem using the post-processing technology, to conduct selected tests in a wind tunnel and analyze the obtained results. The obtained results of the tests, which showed a significant impact of the technological parameters of 3D printing and surface treatment methods on the correctness of the representation of real aerodynamic characteristics, were used mainly to analyze the aerodynamic performance of the model, verify the distribution of forces and moments, and evaluate the behavior of the structure in various flight scenarios. The obtained research results, the analysis of the obtained results, and selected tests were used to present important observations and formulate practical conclusions.

## 1. Introduction

The implementation of modern structural materials, particularly composites, in modern aviation (Airbus A-380, Boeing B-787 and JSF F-35) is dictated by their key role in terms of the benefits obtained, not only in the field of weight reduction, which is obvious, but above all, in terms of the aerodynamic performance achieved.

Current research investigations confirm the use of composites, such as carbon and glass fibers or aramid fibers, in increasing the fuel efficiency of the aircraft by reducing aerodynamic drag [1]. In turn, achieving a lower empty weight of implemented components made of composite materials translates into a reduction in the force required to generate thrust, which has a direct impact on the flight characteristics of the object and energy consumption [2,3,4,5,6,7].

For example, in the construction of basic aircraft components (e.g., wings or fuselage) modern composites are used, characterized by high resistance to bending and mechanical loads while maintaining flexibility. It should also be emphasized that the implementation of such materials allows for the design of more aerodynamically advanced aircraft structures, characterized by the minimization of turbulence effects and improved airflow around the structure. In addition, in this case, the process of lowering the wall layer separation in critical regions is also crucial, resulting in the elimination of the adverse effect of wave drag [8,9,10].

Based on the present critical analysis of the literature, it can be noted that the development of modern composite technologies contributes to the durability of aircraft structures, particularly in terms of resistance to corrosion and chemical effects of the operating environment. In turn, the use of modern composite materials has resulted in the ability to reduce the effects of thermal variables on aerodynamic structures, resulting in more efficient flight stability in various atmospheric conditions.

In papers [11,12,13,14,15,16], the authors investigated the use of modern carbon fibers in terms of their impact on the reduction in aerodynamic drag due to the possibility of designing more advanced airframe shapes. Among other things, they reasoned in this regard that the use of composites based on the use of carbon fibers allows for the creation of structures with lower weight while maintaining exceptional mechanical strength, which has a significant impact on the reduction in drag induced at high flight speeds.

In terms of analyzing the impact of modern composite manufacturing processes, such as automated fiber placement (AFP), on the aerodynamic performance of aircraft, it was shown that by using the technique of precise layering of composite materials, it is possible to obtain surfaces with minimal irregularities, which directly translates into reduced turbulence in the boundary layer. Experimental studies discussed in [17] have confirmed that the use of advanced composite technologies can reduce frictional drag by up to 4% compared to the use of traditional materials.

In turn, the authors in paper [18] proved that the implementation of this type of solution not only allows to improve aerodynamic parameters, but, above all, to increase the flexibility of the structure, which translates into a key importance playing an important role during maneuvers in changing weather conditions. In addition, the use of hybrid composite materials contributes to a more efficient management of the distribution of lifting forces in the different parts of the airframe.

In turn, the work in [19] proves that recycled materials, despite slightly lower mechanical parameters, are used in auxiliary components, which has a considerable impact on reducing the weight of the aircraft. As a result, the economic use of composites leads to an improvement in aerodynamic performance and a reduction in CO_2_ emissions over the product’s life cycle [20].

For instance, in the article [21], the authors carried out an in-depth analysis of the use of composite materials in terms of the structural performance of modern aircraft, with a particular focus on their impact on aerodynamic properties. The key objective of the research inquiry was to evaluate the effectiveness of the use of composite materials in terms of weight reduction and increased aerodynamic efficiency, and thus, improved structural strength of aircraft.

Regarding the use of composite materials (carbon and aramid fibers), significant improvements in aerodynamic performance were observed in terms of aircraft weight reduction and reduced drag, resulting in improved energy efficiency [22,23,24]. Among other things, it was highlighted that the use of composites allows the design of more aerodynamic surfaces while enhancing structural integrity. It was pointed out that aircraft structures made from composites have greater flexibility in the context of design, allowing for the creation of more advanced shapes and aerodynamic profiles. Hence, composite materials are known for their high resistance to corrosion and fatigue, which has a direct impact on the process of extending the aircraft’s life cycle and reducing operating costs.

With regard to the impact on aerodynamics, it has been noted that the integration of composite materials into key structural components (e.g., wings, fuselage, etc.) enables the reduction in turbulence phenomena, resulting in aircraft achieving higher cruise speeds with lower fuel consumption. In addition, detailed analysis of numerical data has also indicated a potential reduction in the negative phenomenon of shock wave generation during supersonic flight as a result of the use of variable stiffness composites [25,26,27]. The results suggested the possibility of optimizing aerodynamic performance based on adapting the composite structure to changing operational conditions. However, it should be noted that a potential challenge remains the cost of manufacturing composite materials, which currently limits their full application in commercial aviation.

Moreover, the use of advanced hybrid composite materials allows the integration of a process of stiffness with flexibility, resulting in a dynamic adaptation of the aircraft structure to changing flight conditions. The research indicates that implementing the use of hybrid composites achieves an optimal balance between aerodynamic stability and stress absorption capability. The authors in this regard have paid particular attention to the effect of such materials on reducing the risk of structural deformation during flight and on improving comfort for passengers [28].

It should also be emphasized that the problem of recycling composite materials in terms of aeronautical applications is of crucial importance. One article discusses the use of recycled carbon fibers and their impact on the aerodynamic performance of an aircraft. It was found that the use of recycled materials not only favors environmental aspects but also imparts structural stiffness without significantly compromising aerodynamic performance [29,30].

In their research, the authors have highlighted that composites allow for a more precise design of aerodynamic shapes, while their elastic and strength properties make it possible to create structures with high resistance to operational deformations. Furthermore, they allow for more efficient management of airflow around the fuselage and wings, which in turn, optimizes lift and minimizes aerodynamic drag [31,32,33,34].

The use of composite materials for aviation also shows an impact on the long-term durability of aircraft structures. In a study carried out in 2021, it was shown that, compared to traditional materials, such as aluminum and steel, composite materials have greater resistance to corrosion and varying environmental conditions such as temperature extremes. It should be noted that this is particularly important in terms of maintaining the aerodynamic performance of the aircraft throughout its lifetime [35].

In the aspect of the problem considered in this article, it should be noted that any airflow in which an object is immersed undergoes a breakup. As air approaches the wing, it sticks to the leading edge, separates, and eventually separates from it to continue flowing along the profile. Specific details of the dynamic air movement around the wing determine the aerodynamic parameters of the wing, which can be studied using computational programs, flight tests, and wind tunnel measurements, which is the subject of this study. Among the aerodynamic properties that are critical to the performance of an airplane are the pitch of the lift curve, the maximum lift, and the angle of attack at which this maximum occurs. The pilot of an airplane can increase the lift in three ways: by increasing the angle of attack α, increasing the speed, and changing the geometry (e.g., by unfolding the blades or flaps) [36].

Aerodynamic tests are carried out on half-models, whole models, or directly on real flying objects (e.g., in-flight tests of airplanes with appropriately mounted sensors and recording equipment). The basic device for laboratory testing is a wind tunnel, in which the essence of the test is to place the test subject in a tunnel with a homogeneous stream of air, with modern wind tunnels usually equipped with platforms that position the model during testing. The purpose of this type of improved dynamic test is to ensure that the complex behavior of an airplane model in a wind tunnel can be observed under different conditions [37].

This manuscript, confirming the crucial importance of FDM-printed model surface quality for the accuracy of obtained results, presents an experimental study of the effect of surface post-processing technology on the aerodynamic characteristics of a selected, 3D-printed M-346 Master supersonic aircraft model. The research problem is clearly formulated to determine how the model’s surface treatment affects the measured results of key aerodynamic parameters. The main idea of this problem is to ensure the accuracy and precision of aerodynamic tests, which play a crucial role in the aviation industry and scientific research.

Based on the above, particularly the research problem and questions, as well as existing theoretical knowledge supported by a critical analysis of the literature, a research hypothesis was formulated assuming that appropriate surface treatment techniques would significantly improve the accuracy of the representation of key aerodynamic parameters of the test object. The main aim of the research was to confirm this hypothesis based on conducting a series of detailed experimental tests. To achieve this aim, a detailed plan was developed based on the 3D printing technique of a model of the M-346 Master military supersonic aircraft and its precision surface treatment.

Aerodynamic tests were carried out based on the M-346 Master aircraft model, using FDM (Fused Deposition Modeling) technology with the PLA (polylactide) material, resulting in the possibility to carry out a series of wind tunnel tests. The results of the tests performed confirmed that the high surface quality of the selected model is a key factor influencing the reliability and accuracy of the measurement results obtained. When measuring the relevant aerodynamic parameters, key differences were observed in the aerodynamic characteristics obtained, depending on the approach used to select the surface treatment of the model under test. In turn, an in-depth analysis of the obtained results allowed both a reliable interpretation of the individual post-processing steps and the final aerodynamic quality of the tested model.

The results obtained clearly and unambiguously confirmed the research hypothesis adopted, highlighting the main differences in the range of selected aerodynamic parameters, the quality of which is dependent on the selected quality of the surface treatment of the model under study. The in-depth analyses made it possible to define in detail the impact of the various stages of the post-processing technology used on the target aerodynamic quality of the model under consideration. The final conclusions and observations based on the conducted research reflect practical application, ensuring quality in terms of streamlining prototyping and model testing procedures not only in aviation, which is the subject of this article, but also in related engineering areas.

The rest of the paper is organized as follows: Section 2 analyses the materials used and the methods employed in terms of the supersonic aircraft model developed for wind tunnel testing. Section 3 is devoted to the obtained test results and their analysis, including a statistical analysis of the sample test results obtained. The development of test results in the case of drag force and lift force was made for different speeds at a constant sideslip angle. Section 4 is devoted to the discussion of the obtained results deals with the obtained measurement results and their analysis. Finally, Section 5 summarizes the work and formulates practical conclusions.

## 2. Materials and Methods

### 2.1. Selection of the Research Object

In modern measurement systems, and thus in the one used for the purposes of this work, the automation of measurements is used, supported by computer techniques necessary for recording, processing, storing, as well as the elaboration and visualization of results [38].

**Figure 1:**
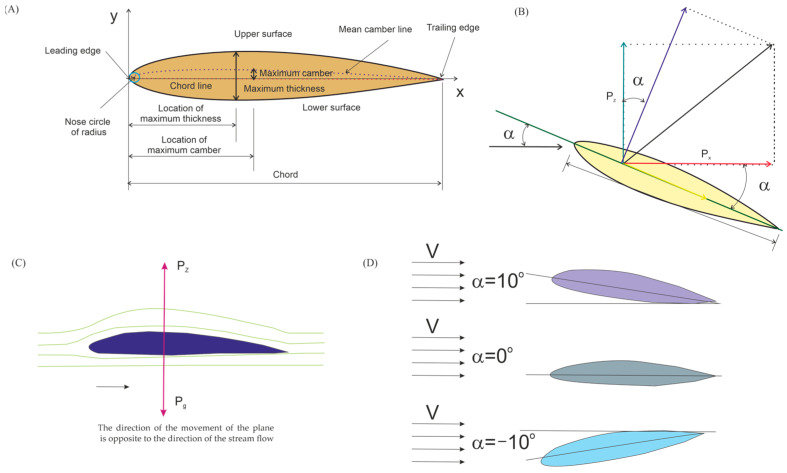
Aerodynamic parameters and phenomena characterising the aircraft [17]: main parameters characterising the airfoil geometry (**A**); aerodynamic forces acting on the airfoil (**B**); [5] aerodynamic forces acting on the airfoil (**C**), and angle of attack of the wing (**D**).

The figure below (Figure 1) shows the key aerodynamic parameters and phenomena characterizing the aircraft, where the following are presented: main parameters characterizing the airfoil geometry (A); aerodynamic forces acting on the airfoil (B); the post-airflow around the wing airfoil (C); and angle of attack of the wing (D) [39,40].

The main force keeping the aircraft in the air is the lift force ($P_{z}$), generated by the wings and acting on the airfoil perpendicular to the airspeed vector in the plane of its symmetry, while the propulsion system provides the thrust force that counteracts air resistance and allows the object to move forward. The factors influencing the profile are the wing surface and air density, which depends on pressure, temperature, and humidity [41,42]. The lift force ($P_{z}$) generated by the wing profile due to the pressure difference is one of the key forces acting on a fixed-wing aircraft, alongside thrust force, drag force ($P_{x}$) and gravity force ($P_{g}$).

The main aerodynamic forces acting on the airfoil are drag $P_{x}$ and lift $P_{z}$. The lift force $P_{z}$ results from integrating the pressure distribution along the airfoil contour (Figure 1B). Airfoil drag, in addition to pressure forces, also includes friction. It is directed along the direction of the unperturbed velocity vector (far from the airfoil) and always points in the same direction as the velocity, while the lift force $P_{z}$ is directed perpendicularly to it and can assume both upward and downward directions.

The lift force $P_{z}$ and drag force $P_{x}$ are components of the aerodynamic force, the value and direction of which depend on the position of the wing in relation to the air streams flowing around the airfoil. The parameter characterizing this setting is the angle of attack ∝, which is contained between the direction of the air stream velocity vector and the chord of the profile. When the chord of the profile is parallel to the direction of the air stream, the angle of attack is zero. When the nose of the profile is higher in relation to the blade, the angle of attack is positive; such an arrangement causes an increase in the lifting force. In the case when the nose is lower than the profile edge, the angle of attack becomes negative. The above-described dependencies are presented in Figure 1D) [43].

**Figure 2:**
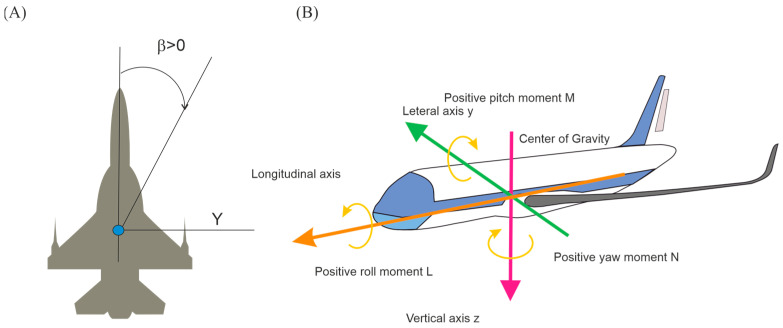
Selected aerodynamic parameters of the test object: sideslip angle (**A**); moments of force acting on the aircraft (**B**).

Figure 2 below shows selected aerodynamic parameters, such as sideslip angle and moments of force of the test object, which are necessary for further analysis.

The measurements in the tunnel can be made not only for the variable angle of attack α, but also depending on the sideslip angle β; in this work, the tests were limited to a constant sideslip angle. This angle is between the velocity vector and the plane of symmetry of the airplane (Figure 2A) [44]. A sideslip angle other than zero causes asymmetric flow around the plane, resulting in a lateral force ($P_{y}$) acting on the plane.

The value of the lift $P_{z}$ that was measured during the tests is defined by the following formula:

(1) $$P_{z}=C_{z}\frac{\rho V^{2}}{2}S,$$

where $C_{z}$—lift force coefficient, determined mainly empirically; ρ—medium density $kg/m^{3}$; V—body velocity in relation to fluid m/s; S—wing area $m^{2}$.

The value of the drag force $P_{x}$ is described by the following formula:

(2) $$P_{x}=\;C_{x}\frac{\rho V^{2}}{2}S,$$

where $C_{x}$—drag force coefficient [45].

The lateral force can be described analogously:

(3) $$P_{y}=\;C_{y}\frac{\rho V^{2}}{2}S,$$

From the calculated lift and drag coefficients, one can obtain another parameter describing the aeronautical profile, i.e., aerodynamic efficiency. It is the ratio of the lift force to the drag force [46,47,48].

Lateral force and lateral moments are regulated by changing the position of controls, such as ailerons or rudder. Positive steering deflection angle is when it causes a negative moment, i.e., right aileron down, left aileron up, rudder left. The occurrence of asymmetrical thrust or geometric asymmetry of the airplane may cause changes in the lateral moments [49].

The aforementioned aerodynamic moments can be described by the following Formulas (4)–(6). Yawing moment:

(4) $$N=C_{n}\frac{\rho V^{2}}{2}Sl,$$

where $C_{n}$—the yaw moment coefficient; ρ—fluid density $kg/m^{3}$; V—body velocity in relation to fluid m/s; S—wing area $m^{2}$; l—wingspan m.

Roll moment:

(5) $$L=C_{l}\frac{\rho V^{2}}{2}Sl,$$

where $C_{l}$—roll moment coefficient.

Pitch moment:

(6) $$M=C_{m}\frac{\rho V^{2}}{2}Sb_{a},$$

where $C_{m}$—the pitch moment coefficient; $b_{a}$—average aerodynamic chord.

The choice of the M-346 Master aircraft model was mainly determined by its aerodynamic characteristics, high maneuverability, and the training aspect in relation to military aviation in the field of technological breakthrough (F-16, JSF F-35).

Based on the above, it was decided to object model in the form of the M-346 Master aircraft, which is an aircraft equipped with state-of-the-art operational systems, as a result of which it is characterized by high maneuverability and the ability to fly even at very high angles of attack, which makes it an excellent training aircraft.

The M-346 Master plane was produced by the Leonardo concern (formerly Alenia Aermacchi-Finmeccanica). The Polish Air Force acquired 8 copies of this modern aircraft with a complete training system in December 2016. Thanks to this purchase, Poland can independently train future pilots of combat aircraft, such as the F-16 and ultimately the F-35. In 2018, they were officially named “Bielik” [50].

The following table (Table 1) summarizes the key parameters of its version, on the basis of which the model under study, which is the subject of consideration in this paper, was created [51].

### 2.2. Preparation of the Aircraft Model

#### 2.2.1. The Technology of 3D Printing

The M-346 Master model was printed on a MakerBot Replicator Z18 3D printer. It is a printer that uses FDM technology, which consists in melting the working material and then applying it with thin threads one on the other. As it solidifies, the material is bonded together, thus creating a hard and durable coating.

The printer is equipped with a heated and closed working chamber inside which a constant temperature is kept in order to minimize material shrinkage. The combination of a sealed chamber with a platform equipped with auto-calibration ensures high quality of the printed model. The relatively large working space of the device (305 × 305 × 457 mm) allows for printing objects of considerable size or even printing several models at the same time. Selected printer parameters are listed in Table 2 [53].

#### 2.2.2. Implementation of a Physical Model

**Figure 3:**
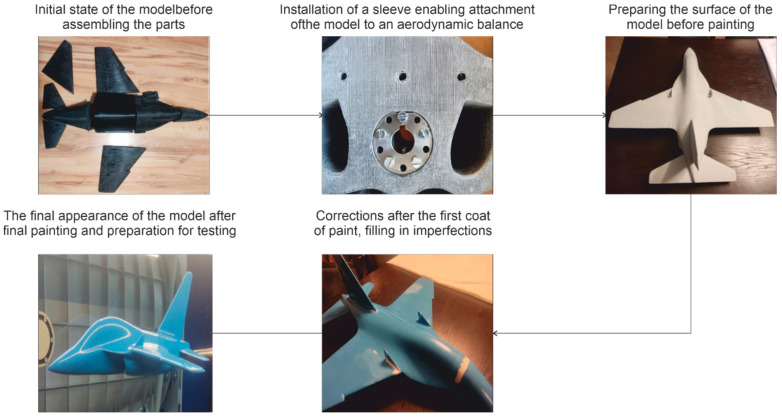
Block diagram of the assembly process according to post-processing technology.

The finished 3D model is exported to the 3D-MakerBot Print printer control software. Before printing, the aforementioned software divides the model into layers with a thickness of 0.2 mm, and then generates a print preview and an executive file for the printer. The first stage of printing is printing the platform that connects the printed parts. The next step is building the object layer by layer according to the programmed shape. The model was printed on a scale of 1 to 11 in 10 elements for post-assembly, which is one of the realizations of the post-processing technology used (Figure 3).

The main purpose of the preparation of the model for measurements was to smooth the elements after printing, as this could have a direct impact on the obtained measurement results. The first stage of the work was the initial smoothing of the surface discontinuities of the elements—transverse depressions characteristic for 3D printing, which cause a rather high undesirable roughness.

In the next stage, the surfaces connecting the model elements and the holes for the reinforcing and fixing rods were cleaned. It was necessary to grind the hole for the sleeve holding the model to the aerodynamic scales, so that it was possible to remove the sleeve, which can be seen in Figure 3. The model was degreased again and painted with two layers of acrylic primer and left to dry for several hours, and then the model was matted with P320 graded paper.

After such preparation of the model, it was possible to cover it with varnish. For this purpose, the model was degreased again and a blue varnish was applied. After drying, further joint imperfections and water stains became visible, which had to be corrected with the use of acrylic mass and P1200 waterproof sandpaper.

In the next stage, the model was re-painted with several layers of blue varnish, and then it was left to dry for 12 h. Then, in order to obtain high smoothness, the test object was painted with two layers of clear varnish. After the last varnish layers had hardened, the model was ready for testing (Figure 3).

#### 2.2.3. Research Stand

**Figure 4:**
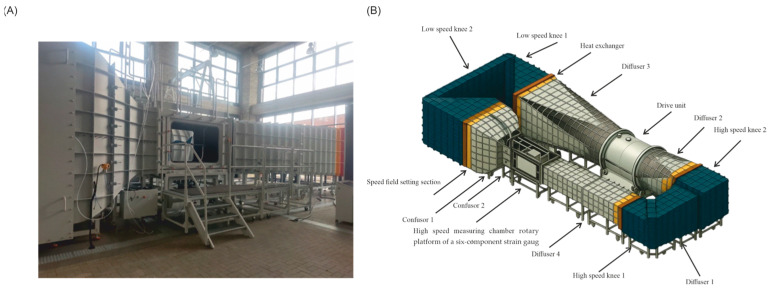
Schematic diagram of the test stand and wind tunnel: test stand (**A**); wind tunnel (**B**).

The tests were carried out in the wind tunnel of the Lublin University of Technology. It is a closed-circuit tunnel with an enclosed measurement space with a rectangular cross-section. The value of the relative turbulence level in the wind tunnel measurement chamber (high-speed chamber) is approximately 0.6–0.8% for a speed range of 20–40 m/s, according to the manufacturer’s specifications and calibrations carried out. The view of the stand is shown in Figure 4A [54].

Figure 4 shows a diagram of the test stand with a description of its individual elements. The structure of the tunnel has been designed in such a way that it is possible to change its configuration by changing the segments. It is possible to use a low and high-speed measuring chamber. A high-speed chamber was used during the tests [54].

The tunnel has been equipped with internal elements shaping the airflow velocity distribution, inspection openings, and manholes allowing for inspection and cleaning of the tunnel interior. The technology used to make and connect the tunnel elements guarantees easy assembly and disassembly, and at the same time, tightness of connections, thus preventing air leakage in the tunnel. Such a solution also prevents the occurrence of faults in the places where elements are joined, which may cause an increase in resistance and losses as well as an increase in the intensity of turbulence in the tunnel.

The proposed wind tunnel at the Lublin University of Technology is primarily intended to conduct comprehensive tests of the effects on various objects, where an object surrounded by air moving at a controlled flow speed is mounted in a measuring chamber. Aerodynamic forces in the form of lift and lateral force ($P_{z}$ and $P_{y\;}$) are generated by the impact of airflow on the surfaces of the tested model. The tested model, placed in the tunnel, is mounted on a special holder connected to measuring sensors that precisely record forces acting in various directions: vertical (lift), horizontal (drag), and lateral (e.g., in cross-winds). During the measurements, data is collected and analyzed in real time using a computer. The lift force $P_{z}$ is the component of the force directed perpendicular to the airflow direction, while the lateral force $P_{y\;}$ can be measured when the air acts at an angle to the model’s location. This type of measurement allows us to see how an object moves, for example, when cornering or in cross-winds. Furthermore, a measurement system installed in a wind tunnel allows for precise testing of vehicle models, structural components, sportswear, and more.

The center of the aerodynamic scales (reference point of the measured forces) is located in the center of rotation of the positioning system. The measuring system works in automatic mode, setting the position of the model on the basis of the prepared grid of angles. The VIBROSON ŁODŹ axial fan is responsible for the tunnel drive, with the technical data presented in Table 3 [54].

**Figure 5:**
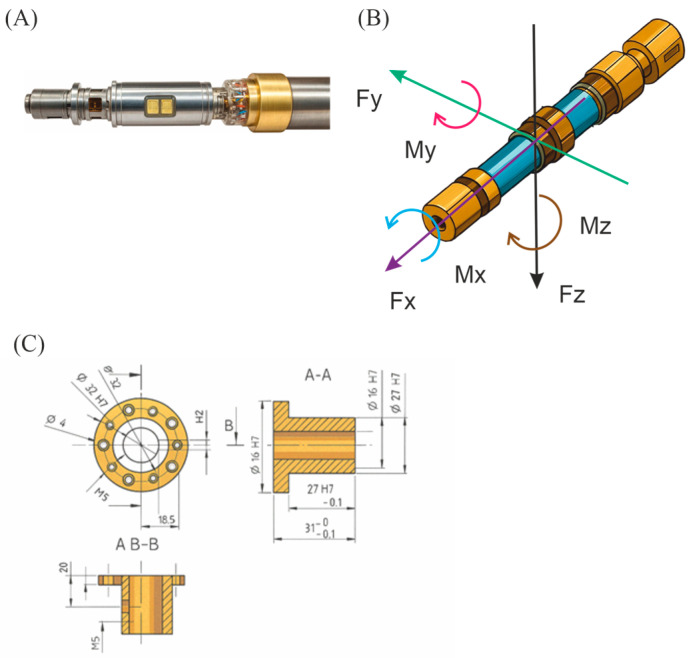
Selected test components: aerodynamic scales mounted in the positioning system (**A**); the coordinate system of the aerodynamic scales (**B**); a drawing of a sleeve mounting a model with aerodynamic scales (**C**).

Measurements of forces and moments in this tunnel are carried out automatically using the FMT 618-1b transducer, which is a “bending beam” transducer. It is made of duplex 17-4 steel with a hardness of approx. 40 HRC [54] (Figure 5A).

Figure 5B shows the coordinate system of the aerodynamic scales. Referring to this system, it is possible to determine the direction of the force or torque by analyzing the measurement results [54]. In Table 4 lists the measuring scopes of individual parameters measured by the aerodynamic scales used for the tests [54].

Figure 5C shows a technical drawing of a sleeve; thanks to this, it was possible to connect the model with an aerodynamic scale [54].

#### 2.2.4. Aerodynamic Research

Before starting the tests, the strength of the model had to be checked to avoid very costly damage, e.g., to the aerodynamic scales or the tunnel. For this purpose, the model was mounted on a dummy of an aerodynamic scales and a test without measurements of forces and moments was simulated. In addition, the tunnel was secured with a net located behind the measuring chamber, which was to protect the tunnel against possible model elements that could be damaged during the test.

After switching on the tunnel, a grid of measuring points was created (α from −20° to +20° every 4°) and the flow velocity was set to 20 m/s. After passing the test, the flow velocity was increased to 30 m/s. At a speed of 40 m/s, the test was carried out with a variable angle of attack but a constant sideslip angle of 0 degrees for safety reasons.

**Figure 6:**
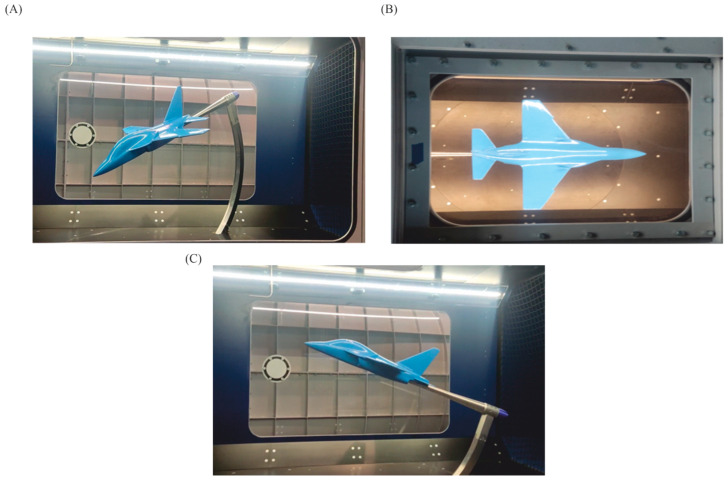
Aircraft model using post-processing technology: during the test at α=−20° and β=0° (**A**); top view of the aircraft model in the measurement chamber (**B**); during measurement at 20 m/s (**C**).

After the successful completion of the test, the main research began. Figure 6A shows the airplane model during the test in the extreme position.

In the next stage, the dummy scales were replaced with a strain gauge transducer, the model was reattached, and the measurements of forces and moments began. For measurements with a flow velocity of 20 m/s and 30 m/s, the test was carried out with the angle of attack changing every 4° in the range from +20° to −20°. On the other hand, measurements at higher flow velocities, i.e., 35, 38, and 40 m/s, were made only with the angle of attack changing from −20° to +20°, but the resolution of the grid of points was reduced to 2°.

**Figure 7:**
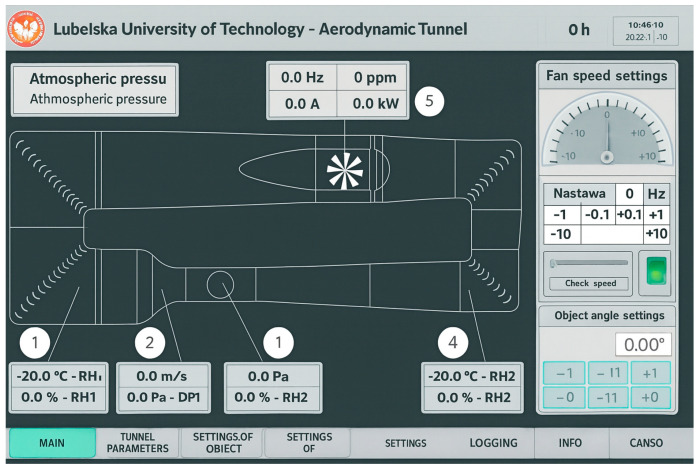
View of the wind tunnel control panel.

Before each measurement session begins, a “dynamic tare” reference measurement is performed for a predetermined grid of measurement points. This measurement allows to determine the influence of forces (mainly gravity) and moments on the model without aerodynamic loading. The signals measured in this way are automatically subtracted from the values obtained during measurements with the aerodynamic loading of the model. Photographs illustrating the model during measurement are shown in Figure 6B,C. The flow velocity in the wind tunnel is set from the operator’s panel (Figure 7) and controlled automatically [54].

On this panel, you can observe a number of parameters in the tunnel in real time, i.e., flow velocity and pressure in the measuring chamber, velocity and pressure difference in the confusor, air temperature and humidity in front of the measuring chamber, temperature and humidity behind the measuring chamber, as well as voltage, intensity, the rotational speed, frequency, and power of the turbofan engine.

**Figure 8:**
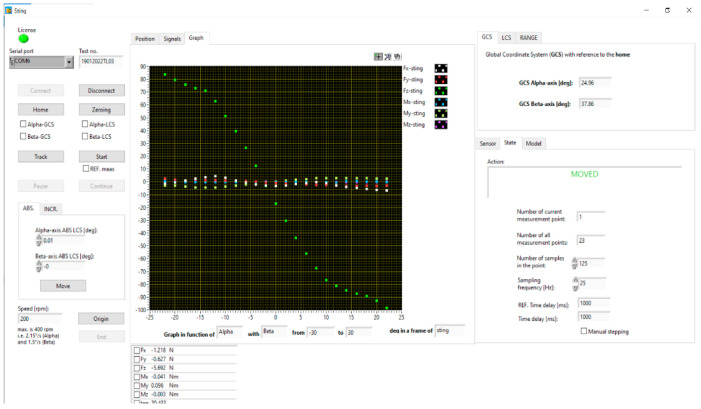
Sting software.

The measurement of forces and moments is carried out using the Sting software, version 2.244.5, which controls the measurement and records the measurement data. The measurement results are displayed in the program in the form of a graph. A file with compiled measurement data for further analysis is saved on the computer’s hard drive. The Sting screenshot is shown in Figure 8 [54].

The tests proceeded without complications, but to maintain safety, the measuring chamber was carefully observed during the measurement, standing close to the safety button, so that the tests could be stopped at any time in the event of, for example, damage to the model. The challenge was the very high current consumption of the axial fan drive. It was necessary to increase the flow speed very slowly so as not to exceed the contractual capacity agreed with the energy company. Ultimately, the assumed flow velocity of 40 m/s was achieved [55]. The elaboration of the results obtained during the measurements and their analysis can be found in the next chapter cited later in this article.

### 2.3. Research Methodology

A detailed analysis of the experimental results, including the methodological approach to preparing the aerodynamic model, was a key element of this article. As a first step, the methodology for the preparation of the aerodynamic model was carefully implemented, including the careful selection of the appropriate 3D printing method involving FDM technology using PLA material.

This resulted in an optimal compromise between high print accuracy and the target production cost of the selected model. Another challenge was the precise surface treatment of the model, including sanding, primer application, and final paint application. In doing so, each step of the post-processing technology used needed to be thoroughly characterized and documented, enabling subsequent analysis of the impact of these activities on the model’s aerodynamic properties.

Aerodynamic tests were carried out in a wind tunnel for five flow speeds, i.e., 20, 30, 35, 38, and 40 m/s and angles of attack (α) ranging from −20° to +20°, with precisely controlled experimental conditions. The subject of the research included measurements of both key aerodynamic forces, such as lift force $P_{z}$, aerodynamic drag $P_{x}$, and lateral force $P_{y}$, and aerodynamic moments in the form of pitching moment M, roll moment L, and yaw moment N.

For this purpose, measuring systems that allowed precise recording of results over a wide range of flow speeds and different angles of attack α were used. The experimental results obtained were presented in tabular and graphical form to make the waveforms of the measured parameters visible, which enabled an in-depth analysis of the data obtained.

When analyzing the results obtained, a clear impact of surface quality on the aerodynamic performance of the model was found. From these results, a significant improvement in the aerodynamic performance of the model was observed after precision surface treatment, particularly in terms of reducing aerodynamic drag and increasing flow stability around the model surface. Furthermore, specific surface treatment steps with the greatest impact on improving aerodynamic performance were identified. Significant differences were also observed between the aerodynamic performance of the model before and after post-processing, confirming the previously formulated research hypothesis.

Taking into account systematic and random errors resulting from the measurement process, particular attention was paid to the analysis of measurement uncertainty. Statistical analysis techniques were used to assess the reproducibility of the results and to determine confidence intervals for the data obtained, with the result that it was possible to confirm the high quality and reliability of the experimental results obtained.

Ultimately, the adopted research methodology and a thorough analysis of the results led to practical conclusions related to the preparation of an aerodynamic model for experimental testing. This approach could become a standard procedure in future experimental aerodynamic studies and serve as a basis for the further development of aerodynamic research methodology. The results obtained can be used in practical applications, especially in aerospace, contributing to improved prototyping and testing of new aerodynamic structures.

## 3. Test Results and Analysis

### 3.1. Development of Test Results at a Constant Sideslip Angle

The next stage of work was to read the recorded values of measured parameters from a file saved by the measuring software. Table 5 shows the results of the drag force and the lift force for different speeds and positive angles of attack.

Table 6 presents the results of measurements of the same parameters, for the same speeds, but for negative angles of attack.

Based on Table 5 and Table 6, the characteristics of drag force $P_{x}$ and lift force $P_{z}$ were drawn up at wind tunnel airspeed for V = 20 m/s, 30 m/s, and 35 m/s, respectively. Similarly, based on the parameters in Table 5 and Table 6, representing the results of measurements of drag force and lift force as a function of angle of attack, which varied from −20° to +20°, were carried out for speeds of 38 m/s and 40 m/s.

**Figure 9:**
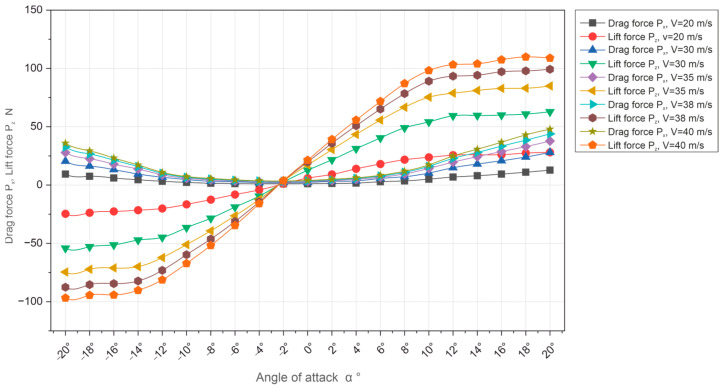
Comparative characteristics of the $P_{x}$ and $P_{z}$ coefficients as a function of the angle of attack ∝ for five air flow speeds (20, 30, 35, 38, and 40 m/s).

Figure 9 shows the collective characteristics of the $P_{x}$ and $P_{z}$ coefficients as a function of the angle of attack ∝ for five speeds.

In Table 7 and Table 8, the magnitude of lateral forces acting on the airplane during aerodynamic tests are shown.

During the tests, the roll and yaw moments were measured, L and N, respectively, the values of which are listed in Table 9 and Table 10.

The above figure shows the collective characteristics of the $C_{x}$ and $C_{z}$ coefficients as a function of the angle of attack ∝ for five speeds.

**Figure 10:**
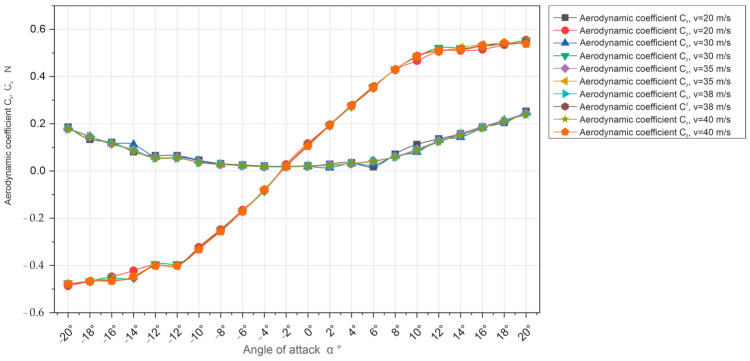
Comparative characteristics of the $C_{x}$ and $C_{z}$ coefficients as a function of the angle of attack ∝ for five air flow speeds (20, 30, 35, 38, and 40 m/s).

The scope of work also included the measurements of the pitch moment M, the values of which for positive angles are presented in Table 11 above, and for negative angles, in Table 12, and the graphic interpretation in the summary, Figure 10.

Then, using transformed relations (1)–(3) from Section 2, dimensionless coefficients, $C_{x}$, $C_{y}$, and $C_{z}$ were determined. An example of the transformed Formula (1) is shown in Formula (7).

Converting the formula into a drag force coefficient:

(7) $$P_{x}=\;C_{x}\frac{\rho V^{2}}{2}S\;\to \;C_{x}=2\frac{P_{x}}{\rho V^{2}S}$$

where $P_{x}$—drag force recorded by the wind tunnel measurement system for a given angle of attack at a given speed N; ρ—the density of the medium in which the measurements were taken, i.e., the air, which depends on the prevailing temperature and pressure, the air temperature during the measurements was on average 21 °C, and the atmospheric pressure was 998 hPa; for these data, the air density value was read from commonly available tables 1.205 $kg/m^{3}$; V—air velocity around the model during the tests, the tests were carried out for five speeds, which were 20, 30, 35, 38, and 40 m/s respectively; S—wing area, which in the case of the considered model, was 0.21 $m^{2}$.

Equally, the formulas were transformed into coefficients of lift and lateral forces. Calculation of the coefficient $C_{x}$ for the speed V=20 m/s and angle of attack α=4°. The drag force in this case is equal to $P_{x}$ = 1.75 N, hence,

(8) $$C_{x}=2\frac{P_{x}}{\rho V^{2}S}=2\cdot \frac{1.75}{1.205\;\cdot 20^{2}\cdot 0.21}=0.034$$

Calculation of the coefficient $C_{x}$ for the speed V=30 m/s, and the angle of attack α=−12°. The drag force in this case is equal to $P_{x}$ = 6.72 N, hence,

(9) $$C_{x}=2\frac{P_{x}}{\rho V^{2}S}=2\cdot \frac{6.72}{1.205\;\cdot 30^{2}\cdot 0.21}=0.058$$

Calculation of the coefficient $C_{z}$ for the speed V=35 m/s, and the angle of attack α=12°. The lift in this case is equal to $P_{z}$ = 78.78 N, therefore,

(10) $$C_{z}=2\frac{P_{z}}{\rho V^{2}S}=2\cdot \frac{78.78}{1.205\;\cdot 35^{2}\cdot 0.21}=0.500$$

Calculation of the coefficient $C_{z}$ for the speed V=38 m/s, and the angle of attack α=−16°. The lift in this case is equal to $P_{z}$ = −84.50 N, so,

(11) $$C_{z}=2\frac{P_{z}}{\rho V^{2}S}=2\cdot \frac{-84.50}{1.205\;\cdot 38^{2}\cdot 0.21}=-0.455$$

Calculation of the coefficient $C_{y}$ for the speed V=20 m/s, angle of attack α=4°. The lateral force in this case is equal to $P_{y}$ = 1.46 N, therefore,

(12) $$C_{y}=2\frac{P_{y}}{\rho V^{2}S}=2\cdot \frac{1.46}{1.205\;\cdot 20^{2}\cdot 0.21}=0.028$$

Calculation of the coefficient $C_{y}$ for the speed V = 30 m/s, the angle of attack α = −8°. The lateral force in this case is equal to $P_{y}$ = −5.39 N, so,

(13) $$C_{y}=2\frac{P_{y}}{\rho V^{2}S}=2\cdot \frac{-5.39}{1.205\;\cdot 30^{2}\cdot 0.21}=-0.11$$

On the basis of the coefficients calculated in this way, Table 13 and Table 14 containing the coefficient values for various configurations. The values of the coefficients are dimensionless. The sign in front of the value indicates the direction of the force, the negative sign indicates the opposite direction of the force to the axes shown in Figure 2b and Figure 5b.

The calculated values of the $C_{x}$ and $C_{z}$ coefficients are presented in charts which graphically present these coefficients depending on the angle of attack. Figure 10 shows the aforementioned dependence for the air flow velocity in the tunnel equal to 20 m/s, respectively, 30, 35, 38, and 40 m/s.

### 3.2. Statistical Analysis of the Results Obtained

**Figure 11:**
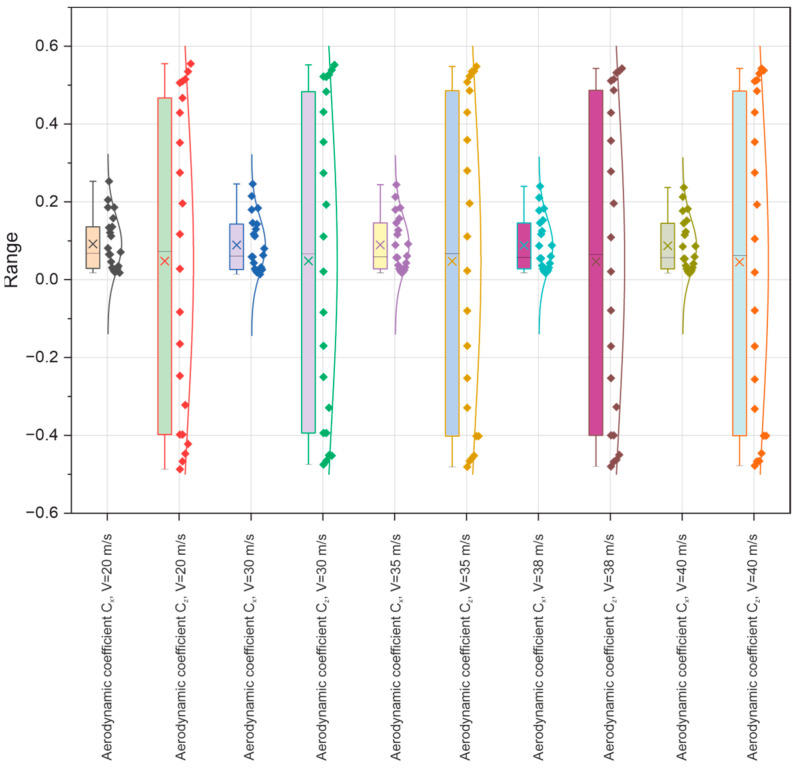
Box plot for the aerodynamic coefficients ($C_{x}$, $C_{z}$) of the tested model.

Based on tests of key aerodynamic parameters in the wind tunnel of the Lublin University of Technology, an in-depth analysis of the significant forces ($P_{x}$, $P_{y}$, and $P_{z}$) and aerodynamic coefficients ($C_{x}$, $C_{y}$, and $C_{z}$) acting on a selected model of the Master M-346 military supersonic aircraft was carried out. The analysis was performed at different airflow speeds (from 20 m/s to 40 m/s), focusing primarily on the influence of the angle of attack ∝ on the distribution of drag forces $P_{x}$ and lift $P_{z}$ and the related coefficients ($C_{x}$, $C_{z}$), as presented in the figure below (Figure 11).

To estimate the scatter and variability of the data obtained in the tests carried out, boxplots were used, augmented with distribution density curves (violin plots) and significant measurement points plotted. The first boxplot illustrates the scatter of data related to the drag force $P_{x}$ and lift force $P_{z}$ relating to the five air speeds (20, 30, 35, 38, and 40 m/s). Analysis reveals that not only the drag force $P_{x}$ but also the lift force $P_{z}$ show increased variability with increasing airflow velocity, primarily for the lift force $P_{z}$ at airflow velocities of 38 and 40 m/s, reflecting greater differences in the values recorded for these velocities. The modification of the box plots by their extension, as well as the existence of outliers, indicates the presence of dynamic changes in the tested aerodynamic characteristics of the aircraft model under consideration, probably resulting from the transition to the state of air stream separation or changes in the local pressure around the aeronautical profile.

**Figure 12:**
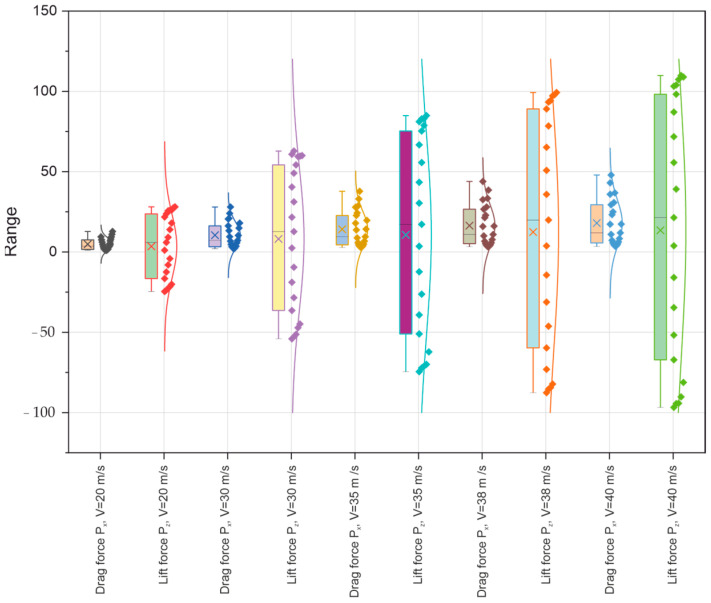
Box plot of aerodynamic forces ($P_{x}$, $P_{z}$) of the model under test.

The second graph (Figure 12) presents an analysis of the dimensionless aerodynamic coefficients associated with the drag forces $P_{x}$ and lift force $P_{z}$, i.e., $C_{x}$ (drag coefficient) and $C_{z}$ (lift force coefficient) for the same five speed values (20, 30, 35, 38, and 40 m/s). It should be noted that the values of the $C_{z}$ coefficients show significant variation; however, for each speed, a cluster of positive values between 0.1 and 0.5 can be observed, indicating the effectiveness of the aerodynamic profile of the model under study in terms of the generation of lift force $P_{z}$. On the other hand, for the $C_{x}$ coefficients, the values range from −0.4 to 0.2, with a greater symmetry towards the zero axis, indicating variation in flows and existing asymmetries in terms of the design or anomalies of the model under research.

Particularly noteworthy are the results for speeds of 35 and 38 m/s, where the largest deviations in $C_{x}$ values were observed, which may be related to the intensity of turbulent phenomena and local variations in the data flow, as well as the identification of outliers which, in terms of aerodynamic testing, may indicate non-linear airflow behavior under specific experimental conditions. This type of approach allows both the identification of critical flow conditions and the possible optimization of the geometry and key design parameters of the model under research.

The results of the aerodynamic analysis of the Master M-346 aircraft model for three selected airflow velocities (20 m/s, 35 m/s, and 40 m/s) made it possible to identify significant relationships between the angle of attack ∝ and the aerodynamic forces and coefficients produced. With increasing speed, a progressive increase could be observed not only in the lift force $P_{z}$, but also in the drag force $P_{x}$, where these values reached a maximum at 40 m/s, 108.9 N and 47.91 N, respectively, indicating that the aerodynamic potential of the airfoil was fully exploited. The highest value of aerodynamic efficiency, in the form of the $C_{z}$/$C_{x}$ ratio, was obtained for an angle of attack value ∝ of +10°, beyond which there was a noticeable decrease in the increase in the lift force $P_{z}$, with a concomitant increase in the drag force $P_{x}$.

The characteristics of the forces and coefficients for each velocity were similar, but at higher velocities (35–40 m/s) the instability and scatter of values increased, possibly indicating a more turbulent flow and a greater susceptibility of the system to small structural asymmetries. In contrast, the values of the lateral force $P_{y}$ and the rolling moments L and pitching moments M also increased with speed, reaching their maxima at angles of attack ∝ between +14° and +18°. The obtained aerodynamic coefficients $C_{x}$ and $C_{z}$ clearly confirmed the correctness of the model and were in agreement with the literature values for similar airfoils.

A key conclusion is also the observed overlap of the $C_{z}$ and $C_{x}$ in the in-path in the mid of the range of angles of attack ∝ (±10°), which demonstrates the consistency of the data and the high quality of the measurements. The results clearly show that the model has the best flight performance in the low and in the moderate range of angles of attack ∝, while in extreme cases, phenomena that may indicate stalling or airflow separation are observed.

## 4. Discussion

### 4.1. Analysis of the Results Obtained

**Figure 13:**
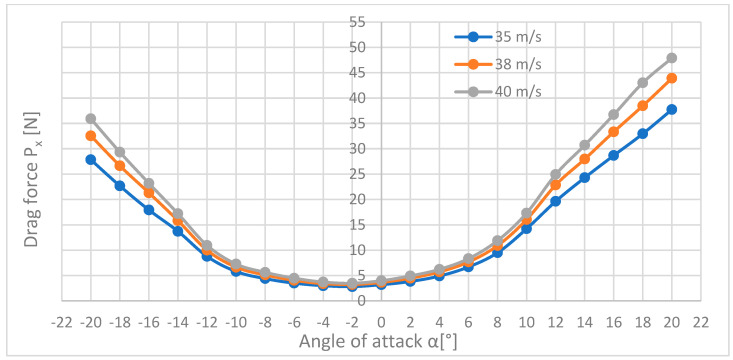
Listing of lateral force coefficients against the angle of attack for three flow velocities.

This subsection analyses the forces, moments, and force coefficients for the three highest speeds, i.e., 35, 38, and 40 m/s, with the tests carried out in the same way for each of these speeds. Table 15 and Table 16 contain the results of calculations of the lateral force coefficient on the basis of Table 7 and Table 8; its waveform is shown in Figure 13.

Figure 13 presents three curves showing the dependence of the drag force on the angle of attack.

Figure 13 shows how the drag forces change when the flow velocity and the angle of attack change. As you can see, the waveforms of forces are almost identical for all three speeds; they only differ in values, but in a narrow range, the angle of attack (from α=−8° to α=+6°) differs little from each other and the difference between them increases with increasing the angle of attack in both directions—negative and positive. The highest drag force was recorded at the speed of 40 m/s in extreme positions; it was the maximum of 47.91 N at an angle of attack of +20°. For negative angles, the drag forces are smaller than for positive angles, which is probably due to the shape of the plane—the top of the plane has a more streamlined shape than its bottom. The lowest drag force is 2.81 N for the speed of 35 m/s and the angle α=−2°.

**Figure 14:**
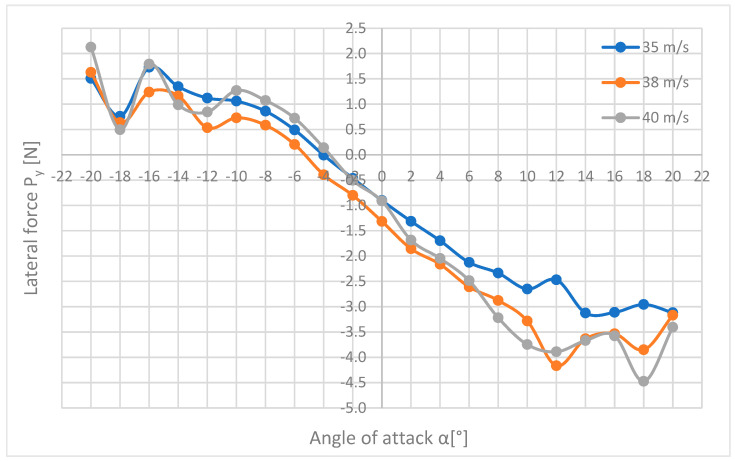
List of lateral force waveforms in relation to the angle of attack for three flow velocities.

Figure 14 shows a summary of the lateral forces $P_{y}$ acting on the M-346 Master model during tests at flow velocities of 35, 38, and 40 m/s.

The diagram (Figure 14) shows the characteristics of the lateral force acting on the plane, which assume small values. The highest lateral force of 4.47 N was obtained for a speed of 40 m/s and an angle of attack of 18°, a negative value informs about the opposite direction of the force to the adopted coordinate system, i.e., in this case, the force acted on the plane from its right side. In the range from −10° to +10° of the angle of attack, the waveforms of the forces are close to linear and increase with an increase in the angle of attack, while for larger angles of attack, this dependence is broken. The lowest forces were recorded for the angle of attack of −4°, where they are close to zero.

**Figure 15:**
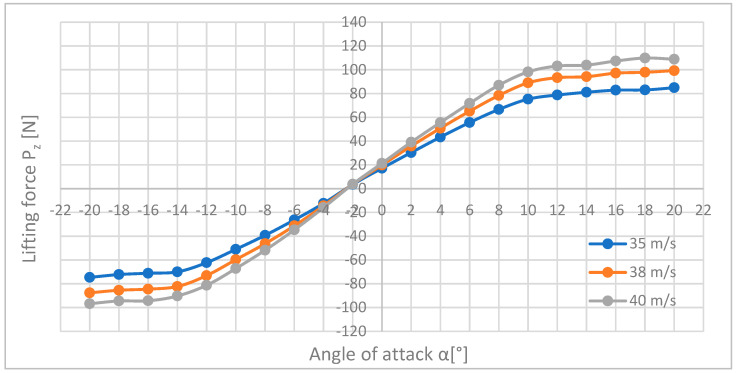
List of the lift force versus the angle of attack for the three flow velocities.

Figure 15 shows the waveforms of the lifting force as a function of the angle of attack for the three maximum flow velocities, i.e., 35, 38, 40 m/s.

The comparative analysis shows that all the lift force waveforms have a very similar shape, which is consistent with the literature standards. Looking at the graph, you can see an upward trend in the lift force along with an increase in the angle of attack α and the speed of the air stream. At an angle of attack equal to 10°, a collapse occurs and increasing the angle of attack does not cause a large increase in the lift force as is the case with smaller angles. The highest lifting force was obtained for a flow velocity of 40 m/s and an angle of attack of +20°; it was 108.9 N.

**Figure 16:**
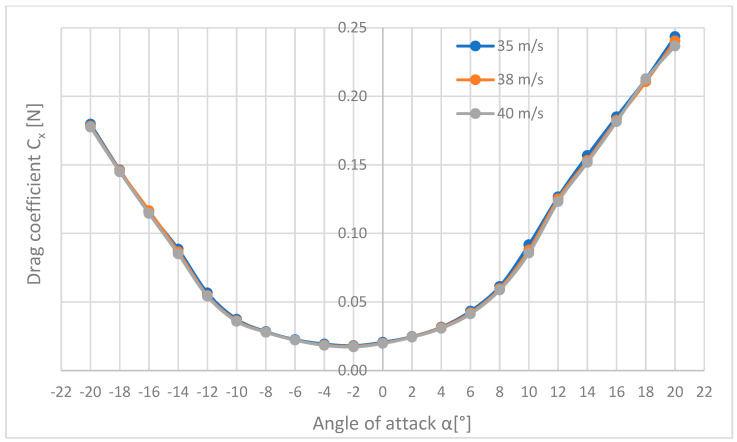
List of courses of coefficients of drag force against the angle of attack for three flow velocities.

**Figure 17:**
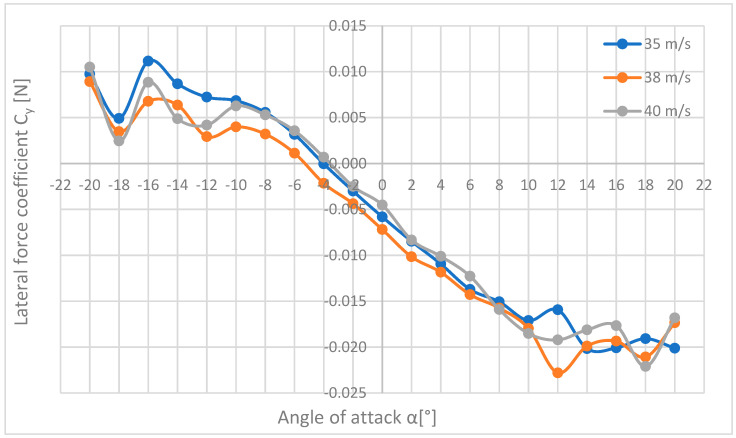
Listing of lateral force coefficients against the angle of attack for three flow velocities.

**Figure 18:**
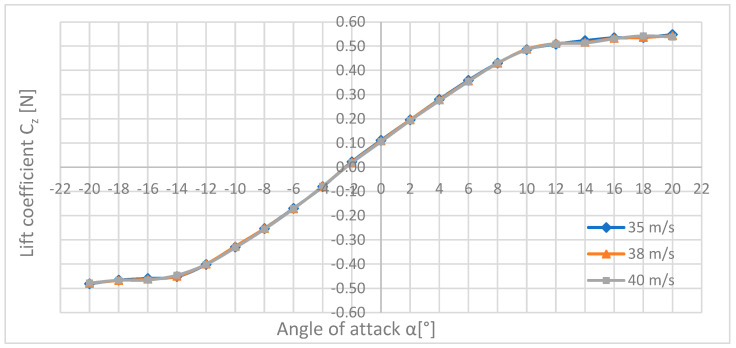
Listing of the courses of lift coefficients versus the angle of attack for three flow velocities.

In the further part of the aerodynamic analysis, a comparison of the drag coefficients $C_{x}$ (Figure 16), drag force $C_{y}$ (Figure 17), and lift $C_{z}$ (Figure 18), calculated earlier in the paper, are presented.

Figure 16 shows that the graphs for all three speeds have identical shapes and take the shape of a parabola with the arms pointing upwards. The curves coincide in the whole range of angles α. The lowest drag force coefficient was obtained for the angle of attack of −2° and it was 0.017 for the speed of 40 m/s, and for the speeds of 35 and 38 m/s, it was almost the same and amounted to 0.018. The highest drag force coefficient was recorded for the speed of 35 m/s and was equal to 0.244.

Figure 17 shows how the coefficients of the lateral force acting on the plane change and what low values are during the tests. For angles of attack α from −20° to +10°, the graphs have a similar shape, while for angles from +10° and above, there are significant discrepancies. The smallest values of the lateral force coefficient occur for the angle of attack α = 4° and are almost equal to 0. The largest coefficient of lateral force is 0.023 and corresponds to the same point as the maximum lateral force, i.e., α = +12°, and the flow velocity V = 38 m/s.

As can be seen in Figure 18, the waveforms of the lift force coefficients $C_{z}$ have the same shape to such an extent that all three curves line up in one line. When analysing the graph, it can be noticed that the values of the coefficient increase linearly in the range of angles of attack α from −12° to +10°; above this point, the increase in the lift force breaks down, which increases only slightly, and may even remain at a constant level. The maximum lift force coefficient $C_{z}$ of 0.548 was obtained for the flow velocity of 35 m/s and the angle of attack α = +20°, while the lowest 0.019 was for the speed of 40 m/s and the angle of attack α = −2°.

**Figure 19:**
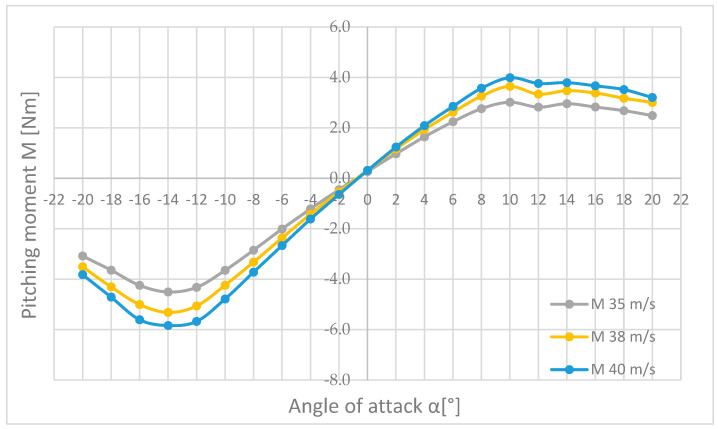
List of the waveforms of the value of the pitch moment M in relation to the angle of attack α for the three flow velocities, 35, 38, and 40 m/s.

**Figure 20:**
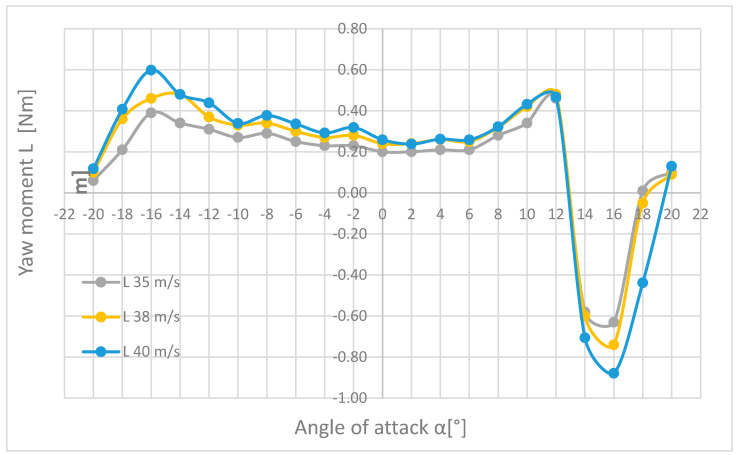
List of the waveforms of the roll moment L in relation to the angle of attack α for the three flow velocities, 35, 38, and 40 m/s.

**Figure 21:**
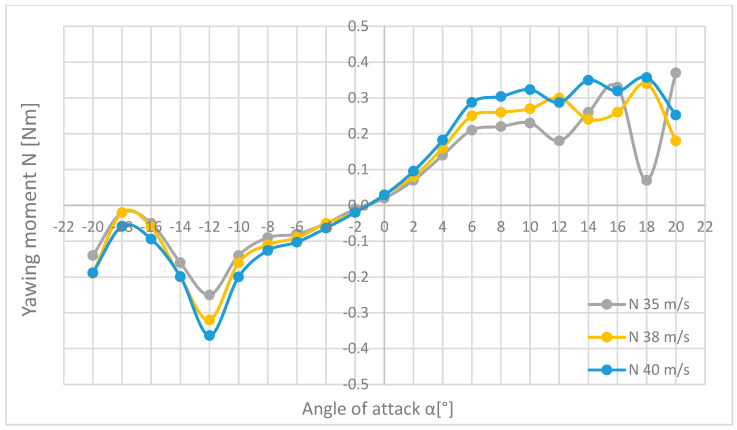
List of the waveforms of the value of the yaw moment N in relation to the angle of attack α for the three flow velocities, 35, 38, and 40 m/s.

The next combinations were prepared for the pitch moment M (Figure 19), the roll moment L (Figure 20), and the yaw moment N (Figure 21), for the flow velocities of 35, 38, and 40 m/s.

In Figure 19 it can be seen that the pitch moment diagrams for all speeds have a very similar shape and differ only in the values depending on the angle of attack and the speed of the air stream in the wind tunnel. The pitch moment M is zero at an angle of attack α equal to about −1°, and as the nose of the aircraft moves upward, it increases until it reaches the angle of attack α = +10° and then begins to decrease until the end of the measurement. In the case of negative angles, it is similar; the moment increases in the opposite direction until reaching the angle of attack α = −14° and begins to decrease significantly, much faster than in the case of positive angles. The maximum pitch moment M is 3.99 Nm for a flow velocity of 40 m/s and an angle of attack α = +10°.

Analyzing the graph in Figure 20, it can be seen that the waveforms of the roll moment L have a similar shape; slight discrepancies occur in a narrow range of angles of attack from −16° to −10°. In the extreme position, where the nose of the plane is down, the moment if it is close to zero and increases to −16°, then slightly decreases until reaching the angle of attack α equal to +6°, where the upward trend begins again. After exceeding the angle of attack equal to +12°, there is a sudden change in the direction of operation and an increase in the roll moment; at +16°, it reaches the maximum value equal to (in the case of a flow velocity of 40 m/s) 0.88 Nm. Then the torque decreases, and for speeds of 35 and 38 m/s, it approaches zero at an angle of attack of +18°, and at an angle of +20°, it is clearly visible that regardless of the flow velocity, the roll moment has similar values.

Looking at Figure 21, it can be seen that the shapes of the waveforms of the values of the yaw moments N are similar to each other, and the range of small angles of attack α even coincide. There are slight discrepancies in the range of the attack angles α from 10° to 20°. It is not difficult to notice the wave of the moment at angles from −20° to −10°, but after exceeding this point, the situation stabilizes and the moment decreases almost to zero until the angle of attack α is reached and equal to −2°, where the direction of the moment action changes and its significant increase up to an angle of attack of +10°. Further increasing the angle of attack causes the torque value to ripple again. The highest yaw moment N was measured for the measurement at a flow velocity of 35 m/s and an angle of attack α = 20°.

During the analysis, it was noticed that increasing the angle of attack α above +10° in order to obtain an increase in the lift force, $P_{z}$ does not make much sense, because when this point is exceeded, the large increase in the said lift force collapses. On the other hand, for the same angle of attack α, there is a large increase in the drag force $P_{x}$, causing a decrease in speed, and at the same time, also the economy of flight, which is highest at this angle of attack α. This is confirmed by the highest ratio of the lift coefficient $C_{z}$ to the drag force coefficient $C_{x}$ for this point; it can be seen, in Figure 19.

The presence of a lateral force $P_{y}$ and a yawing moment N may indicate the lack of symmetry of the airplane with respect to the vertical surface passing through the longitudinal axis of the airplane. This may be due to the accuracy of 3D printing or imperfections in the assembly of the model. On the other hand, undulations in the graphs near the extreme positions may result from the vibrations of the model, which were visible during the tests in these conditions.

A small roll moment L occurring in almost the entire range of angles of attack α may indicate a minimal difference in the mounting of the wings (left and right) in relation to each other, i.e., the wings can assume different angles of attack unnoticed. In this case, a positive moment would indicate a slight lowering of the nose of the right wing profile in relation to the left wing.

### 4.2. Scientific Contribution

It should be emphasized that, in the light of the research carried out, the scientific contribution of the present study is to experimentally not only demonstrate, but also confirm, the effect of surface treatment on the aerodynamic characteristics of the 3D printed aircraft model. The cited test results clearly show that correctly performed surface treatment processes definitely improve the accuracy of the representation of aerodynamic parameters, which in turn, is of crucial importance from the point of view of the reliability and quality of experimental aerodynamic tests.

The development of a comprehensive experimental test plan made it possible to precisely establish the relationship between surface quality and the measurement results obtained. As a result, a scientific basis has been established for the further development and optimization of post-processing techniques that can be implemented in accordance with current standards for prototyping and testing of aerodynamic models used in engineering practice, especially in aviation and related areas. Therefore, this article makes a significant contribution to the development of experimental methodology and provides a basis for further research into improving the quality and precision of aerodynamic prototyping. Hence, the article makes a significant contribution to the development of experimental methodology and provides a basis for further research into improving the quality and accuracy of aerodynamic prototyping.

The original contribution of the present work is the experimental confirmation and in-depth analysis of the impact of the surface treatment quality of the model under study on its aerodynamic performance through the use of 3D printing techniques. As a result, a direct relationship between the application of specific finishing techniques and the accuracy of the obtained measurements of key aerodynamic parameters was comprehensively analyzed and established.

The tests carried out provided the opportunity to pinpoint key steps and surface treatment techniques that clearly improve the representation of the actual aerodynamic characteristics. Hence, the work contributes valuable new data to the field of experimental aerodynamic research, offering specific recommendations for the preparation of test models. The results obtained also allow the further evolution of quality standards for aerodynamic prototyping, finding wide use in the field of scientific research and in industrial practice, particularly in the aerospace industry.

## 5. Summary and Conclusions

The experimental data collected for the research was discussed in depth and the conclusions were compared with the current knowledge in the field of aerodynamics. Aerodynamic tests conducted on a selected supersonic aircraft model, printed using FDM using PLA, allowed for the formulation of a number of important conclusions regarding both the model’s manufacturing technology and the impact of material preparation on the results of the aerodynamic tests.

During experimental studies of the flow visualization of the M-346 Master aircraft model, a change in the flow character was observed with changes in the angle of attack α and the flow velocity. As the angle of attack α increased, vortices were observed to form on the leading edge of the airfoil and wing. The resulting vortices caused the flow to “stick” to the airfoil. Furthermore, a separation pattern was observed on the wing surface of the tested aircraft model.

The obtained results clearly confirm the validity of the adopted research hypothesis and define the direction of further work, particularly in terms of optimizing post-processing methods. Thus, they constitute an important step in the validation of training aircraft models in real-world operating conditions.

The results obtained are widely transferable in terms of aeronautical implications, providing more accurate and reliable prototyping of new aerodynamic solutions. Furthermore, the research results can serve as a basis for evaluating procedures and standards for developing and testing aerodynamic research models.

The research methodology presented in this manuscript can be used in aviation, as well as in other engineering fields where the surface quality of prototypes and models, plays a key role and is characterized by practical applications. As a result, the research carried out not only deepens the existing theoretical knowledge in aerodynamics, but, above all, outlines specific implications for the expansion of practical knowledge resulting from the proper selection of post-processing technologies for the surface of the developed model. Furthermore, the scientific novelty highlighted in the article in the aspect of experimental confirmation of the effect of post-processing of a 3D printed model on aerodynamic characteristics is an important contribution to the evolution of contemporary experimental methods and techniques in aeronautical engineering.

Tests were carried out for five flow velocities, i.e., 20, 30, 35, 38, and 40 m/s, for a range of angles of attack α from −20° to +20°. The results obtained are presented in tabular and graphical form, as well as based on the statistical analysis performed using box plots for the forces ($P_{x}$, $P_{z}$) and dimensionless aerodynamic coefficients ($C_{x}$, $C_{z}$) of the tested model illustrated in Figure 20 and Figure 21, respectively.

In the course of the paper, six key quantities affecting the aircraft during flight were measured in the form of forces, i.e., drag force ($P_{x}$), lateral force ($P_{y}$), and lift force ($P_{z}$), as well as pitching (M), roll (L), and yawing (N) moments. All parameters were analysed, but only at the three highest flow velocities, i.e., 35, 38, and 40 m/s, summary plots (Figure 19, Figure 20 and Figure 21) were created so that an in-depth analysis of the results was possible once the results had been systematized.

Referring to the analysis of the results obtained, it should be emphasized that the tests were performed correctly and the results were probable and consistent with the expectations and reference standards, which is confirmed, for example, by collective graphs of force coefficients, the drag force $P_{x}$ and lift force $P_{z}$, and respectively, i.e., $C_{x}$ and $C_{z}$ (Figure 9 and Figure 10), where the curves for all speeds overlap. The overlapping of the curves on the graph also proves the high measurement accuracy of the aerodynamic balance used.

In conclusion, based on the results obtained, recommendations for future works are the following: continuing to develop methods for preparing the surfaces of 3D printed models to further improve the quality of aerodynamic testing; considering the use of alternative printing technologies and materials for tests requiring higher accuracy; analyzing the effect of model surface roughness on the formation of turbulence and the change in aerodynamic forces as a function of different flight scenarios; and expanding the study to include the effect of different 3D printing technologies on the aerodynamic parameters of models under variable speed conditions for both the angle of attack α and sideslip angle β.

Moreover, it should be added that as part of further work on the issues considered in this article, the authors believe it would be interesting to make research inquiries into the analysis of aerodynamic characteristics not only at variable angles of attack, but also at variable sideslip angles. For example, conducting tests and analyzing the results obtained for five flow velocities, i.e., 20, 30, 35, 38, and 40 m/s, and a range of angles of attack α from −20° to +20° and sideslip angles β also from −20° to +20°.

## Figures and Tables

**Table 1:** Selected parameters of the Master M-346 aircraft [52].

Parameter	Quantity
The span	9.72 m
Length	11.49 m
Height	4.98 m
Bearing surface	23.52 $m^{2}$
Own weight	4610 kg
Take-off weight	6700 kg
Engine type	2 × F124-GA-200
Thrust	2 × 2835 kg
Fuel consumption	50 kg/min
Speed (at height 1524)	1093 km/h
The climb	102 m/s
Ceiling	13,716 m
The run-up	290 m
Landing run (with minimal fuel reserve)	550 m
Range	1852 km
Permissible overload factors for the structure	+8 g/−3 g

**Table 2:** Selected technical parameters of the MakerBot Replicator Z18 printer.

Parameter	Quantity
Material	PLA (Polilaktyd)
Layer thickness	0.1 mm
Material diameter	1.75 mm
Nozzle diameter	0.4 mm
XY positioning accuracy	0.011 mm
Z positioning accuracy	0.0025 mm

**Table 3:** Selected technical data of an axial fan.

Parameter	Quantity
Performance	106 $m^{3}/s$
Piling up	1200 Pa
Rotation speed	594 rpm
Engine power	160 kW
Mass	5850 kg

**Table 4:** Measuring scopes of the transducer.

Parameter	Scope
$F_{x}$	±170 N
$F_{y}$	±350 N
$F_{z}$	±400 N
$M_{x}$	±10 Nm
$M_{y}$	±19 Nm
$M_{z}$	±21 Nm

**Table 5:** Measurement results $P_{x}$ and $P_{z}$ in the wind tunnel for positive angles.

α °	v=20 m/s		v=30 m/s		v=35 m/s		v=38 m/s		v=40 m/s	
	$P_{x}$ N	$P_{z}$ N	$P_{x}$ N	$P_{z}$ N	$P_{x}$ N	$P_{z}$ N	$P_{x}$ N	$P_{z}$ N	$P_{x}$ N	$P_{z}$ N
0°	1.12	5.92	2.27	12.67	3.20	17.20	3.65	19.85	4.00	21.36
2°					3.84	30.37	4.47	35.81	4.92	39.09
4°	1.75	13.91	3.59	31.17	4.91	43.40	5.72	50.82	6.23	55.68
6°					6.71	55.66	7.69	65.14	8.36	71.75
8°	3.58	21.72	7.17	49.05	9.51	66.66	10.90	78.44	11.90	87.05
10°					14.23	75.31	16.06	89.04	17.33	98.19
12°	6.87	25.6	14.85	59.4	19.64	78.78	22.87	93.35	24.94	103.18
14°					24.31	81.10	27.99	94.18	30.72	103.92
16°	9.4	26.07	20.9	59.99	28.68	82.88	33.36	97.17	36.75	107.39
18°					32.97	83.04	38.49	97.90	43.03	109.87
20°	12.78	28.07	27.97	62.84	37.76	84.92	43.92	99.29	47.91	108.90

**Table 6:** Measurement results $P_{x}$ and $P_{z}$ in the wind tunnel for negative angles.

α °	v=20 m/s		v=30 m/s		v=35 m/s		v=38 m/s		v=40 m/s	
	$P_{x}$ N	$P_{z}$ N	$P_{x}$ N	$P_{z}$ N	$P_{x}$ N	$P_{z}$ N	$P_{x}$ N	$P_{z}$ N	$P_{x}$ N	$P_{z}$ N
0°	1.12	5.92	2.27	12.67	3.20	17.20	3.65	19.85	4.00	21.36
−2°					2.81	3.52	3.23	3.76	3.49	3.80
−4°	1.06	−4.19	2.15	−9.56	3.00	−12.38	3.40	−14.38	3.72	−15.94
−6°					3.50	−26.31	4.06	−31.26	4.48	−34.67
−8°	1.56	−12.48	3.21	−28.48	4.40	−39.22	5.12	−46.25	5.64	−51.76
−10°					5.80	−51.03	6.65	−59.72	7.27	−67.18
−12°	3.28	−20.13	6.72	−44.87	8.78	−62.23	10.01	−73.10	10.96	−81.25
−14°					13.72	−69.99	15.84	−82.28	17.22	−90.26
−16°	6.10	−22.64	13.28	−51.33	17.94	−71.13	21.32	−84.50	23.20	−94.25
−18°					22.68	−72.24	26.65	−85.44	29.35	−94.48
−20°	9.39	−24.64	20.50	−54.11	27.86	−74.59	32.57	87.61	35.93	−96.79

**Table 7:** Results of measurement results $P_{y}$ in a wind tunnel for positive angles.

α °	$P_{y}$ N				
	v=20 m/s	v=30 m/s	v=35 m/s	v=38 m/s	v=40 m/s
0°	−0.34	−0.84	−0.90	−1.31	−0.91
2°			−1.31	−1.85	−1.69
4°	−0.59	−1.34	−1.70	−2.16	−2.05
6°			−2.12	−2.61	−2.49
8°	−0.73	−1.89	−2.33	−2.88	−3.22
10°			−2.65	−3.28	−3.75
12°	−0.69	−2.18	−2.47	−4.17	−3.89
14°			−3.13	−3.63	−3.67
16°	−0.92	−2.14	−3.11	−3.53	−3.58
18°			−2.96	−3.85	−4.47
20°	−0.79	−1.70	−3.12	−3.17	−3.40

**Table 8:** Results of measurement results $P_{y}$ in a wind tunnel for negative angles.

α °	$P_{y}$ N				
	v=20 m/s	v=30 m/s	v=35 m/s	v=38 m/s	v=40 m/s
0°	−0.34	−0.84	−0.90	−1.31	−0.91
−2°			−0.46	−0.80	−0.50
−4°	−0.08	−0.12	−0.01	−0.39	0.14
−6°			0.49	0.20	0.72
−8°	0.17	0.49	0.86	0.58	1.07
−10°			1.06	0.73	1.27
−12°	0.23	0.62	1.12	0.53	0.85
−14°			1.35	1.16	0.99
−16°	0.4	0.91	1.73	1.24	1.79
−18°			0.76	0.63	0.50
−20°	0.47	0.47	1.51	1.63	2.13

**Table 9:** Results of measurements of moments L and N in the wind tunnel for negative angles.

α °	v=20 m/s		v=30 m/s		v=35 m/s		v=38 m/s		v=40 m/s	
	L Nm	N Nm	L Nm	N Nm	L Nm	N Nm	L Nm	N Nm	L Nm	N Nm
0°	0.06	0.01	0.14	0.02	0.20	0.02	0.24	0.03	0.26	0.03
−2°					0.23	−0.01	0.28	−0.02	0.32	−0.02
−4°	0.12	0.00	0.19	−0.02	0.23	−0.05	0.27	−0.05	0.29	−0.06
−6°					0.25	−0.08	0.30	−0.09	0.34	−0.10
−8°	0.09	−0.02	0.21	−0.06	0.29	−0.09	0.34	−0.11	0.38	−0.13
−10°					0.27	−0.14	0.33	−0.16	0.34	−0.20
−12°	0.09	−0.05	0.20	−0.12	0.31	−0.25	0.37	−0.32	0.44	−0.36
−14°					0.34	−0.16	0.48	−0.20	0.48	−0.20
−16°	0.11	−0.02	0.37	0.03	0.39	−0.05	0.46	−0.06	0.60	−0.09
−18°					0.21	−0.02	0.36	−0.02	0.41	−0.06
−20°	0.06	−0.01	0.04	−0.11	0.06	−0.14	0.10	−0.19	0.12	−0.19

**Table 10:** Results of measurements of moments L and N in the wind tunnel for positive angles.

α °	v=20 m/s		v=30 m/s		v=35 m/s		v=38 m/s		v=40 m/s	
	L Nm	N Nm	L Nm	N Nm	L Nm	N Nm	L Nm	N Nm	L Nm	N Nm
0°	0.06	0.01	0.14	0.02	0.20	0.02	0.24	0.03	0.26	0.03
2°					0.20	0.07	0.24	0.08	0.24	0.10
4°	0.06	0.04	0.15	0.10	0.21	0.14	0.26	0.16	0.26	0.18
6°					0.21	0.21	0.25	0.25	0.26	0.29
8°	0.09	0.06	0.19	0.16	0.28	0.22	0.32	0.26	0.32	0.30
10°					0.34	0.23	0.42	0.27	0.43	0.32
12°	0.31	0.03	0.27	0.16	0.46	0.18	0.48	0.30	0.47	0.29
14°					−0.58	0.26	−0.60	0.24	−0.71	0.35
16°	0.04	0.06	−0.12	0.19	−0.63	0.33	−0.74	0.26	−0.88	0.32
18°					0.01	0.07	−0.05	0.34	−0.44	0.36
20°	0.04	0.02	0.09	0.05	0.10	0.37	0.09	0.18	0.13	0.25

**Table 11:** Results of measurements of moment M in the wind tunnel for positive angles.

α °	M Nm				
	v=20 m/s	v=30 m/s	v=35 m/s	v=38 m/s	v=40 m/s
0°	0.11	0.21	0.27	0.32	0.31
2°			0.97	1.16	1.24
4°	0.54	1.2	1.64	1.93	2.09
6°			2.24	2.62	2.85
8°	0.92	2.05	2.76	3.26	3.57
10°			3.02	3.64	3.99
12°	0.99	2.17	2.82	3.34	3.76
14°			2.96	3.47	3.79
16°	0.97	2.19	2.82	3.38	3.67
18°			2.68	3.18	3.52
20°	0.83	1.91	2.49	3.01	3.20

**Table 12:** Results of measurements of moment M in the wind tunnel for negative angles.

α °	M Nm				
	v=20 m/s	v=30 m/s	v=35 m/s	v=38 m/s	v=40 m/s
0°	0.11	0.21	0.27	0.32	0.31
−2°			−0.45	−0.54	−0.64
−4°	−0.4	−0.9	−1.21	−1.42	−1.61
−6°			−2.01	−2.36	−2.66
−8°	−0.93	−2.08	−2.85	−3.32	−3.72
−10°			−3.65	−4.24	−4.78
−12°	−1.36	−3.08	−4.32	−5.06	−5.67
−14°			−4.51	−5.32	−5.83
−16°	−1.34	−2.99	−4.24	−5.00	−5.61
−18°			−3.64	−4.31	−4.71
−20°	−1.00	−2.18	−3.08	−3.51	−3.82

**Table 13:** Calculation results of the coefficients $C_{x}$ and $C_{z}$ for positive angles.

α °	v=20 m/s		v=30 m/s		v=35 m/s		v=38 m/s		v=40 m/s	
	$C_{x}$ N	$C_{z}$ N	$C_{x}$ N	$C_{z}$ N	$C_{x}$ N	$C_{z}$ N	$C_{x}$ N	$C_{z}$ N	$C_{x}$ N	$C_{z}$ N
0°	0.022	0.117	0.020	0.111	0.021	0.111	0.020	0.109	0.020	0.105
2°					0.025	0.196	0.024	0.196	0.024	0.193
4°	0.035	0.275	0.032	0.274	0.032	0.280	0.031	0.278	0.031	0.275
6°					0.043	0.359	0.042	0.357	0.041	0.354
8°	0.071	0.429	0.063	0.431	0.061	0.430	0.060	0.429	0.059	0.430
10°					0.092	0.486	0.088	0.487	0.086	0.485
12°	0.136	0.506	0.130	0.522	0.127	0.508	0.125	0.511	0.123	0.510
14°					0.157	0.523	0.153	0.515	0.152	0.513
16°	0.186	0.515	0.184	0.527	0.185	0.535	0.183	0.532	0.182	0.530
18°					0.213	0.536	0.211	0.536	0.213	0.543
20°	0.253	0.555	0.246	0.552	0.244	0.548	0.240	0.543	0.237	0.538

**Table 14:** Calculation results of the coefficients $C_{x}$ and $C_{z}$ for negative angles.

α °	v=20 m/s		v=30 m/s		v=35 m/s		v=38 m/s		v=40 m/s	
	$C_{x}$ N	$C_{z}N$	$C_{x}$ N	$C_{z}N$	$C_{x}$ N	$C_{z}N$	$C_{x}$ N	$C_{z}\;N$	$C_{x}$ N	$C_{z}\;N$
0°	0.022	0.117	0.020	0.111	0.021	0.111	0.020	0.109	0.020	0.105
−2°					0.018	0.023	0.018	0.021	0.017	0.019
−4°	0.021	−0.083	0.019	−0.084	0.019	−0.080	0.019	−0.079	0.018	−0.079
−6°					0.023	−0.170	0.022	−0.171	0.022	−0.171
−8°	0.031	−0.247	0.028	−0.250	0.028	−0.253	0.028	−0.253	0.028	−0.256
−10°					0.037	−0.329	0.036	−0.327	0.036	−0.332
−12°	0.065	−0.398	0.059	−0.394	0.057	−0.402	0.055	−0.400	0.054	−0.401
−14°					0.089	−0.452	0.087	−0.450	0.085	−0.446
−16°	0.121	−0.447	0.117	−0.451	0.116	−0.459	0.117	−0.463	0.115	−0.466
−18°					0.146	−0.466	0.146	−0.468	0.145	−0.467
−20°	0.186	−0.487	0.180	−0.475	0.180	−0.481	0.178	0.480	0.177	−0.478

**Table 15:** The results of calculating the coefficients $C_{y}$ for positive angles.

α °	$C_{y}$ N				
	v=20 m/s	v=30 m/s	v=35 m/s	v=38 m/s	v=40 m/s
0°	−0.007	−0.007	−0.006	−0.007	−0.005
2°			−0.008	−0.010	−0.008
4°	−0.012	−0.012	−0.011	−0.012	−0.010
6°			−0.014	−0.014	−0.012
8°	−0.014	−0.017	−0.015	−0.016	−0.016
10°			−0.017	−0.018	−0.019
12°	−0.014	−0.019	−0.016	−0.023	−0.019
14°			−0.020	−0.020	−0.018
16°	−0.018	−0.019	−0.020	−0.019	−0.018
18°			−0.019	−0.021	−0.022
20°	−0.016	−0.015	−0.020	−0.017	−0.017

**Table 16:** The results of calculating the coefficients $C_{y}$ for negative angles.

α °	$C_{y}$ N				
	v=20 m/s	v=30 m/s	v=35 m/s	v=38 m/s	v=40 m/s
0°	−0.007	−0.007	−0.006	−0.007	−0.005
−2°			−0.003	−0.004	−0.002
−4°	−0.002	−0.001	0.000	−0.002	0.001
−6°			0.003	0.001	0.004
−8°	0.003	0.004	0.006	0.003	0.005
−10°			0.007	0.004	0.006
−12°	0.005	0.005	0.007	0.003	0.004
−14°			0.009	0.006	0.005
−16°	0.008	0.008	0.011	0.007	0.009
−18°			0.005	0.003	0.002
−20°	0.009	0.004	0.010	0.009	0.011

## Data Availability

The data presented in this study are available on request from the corresponding author.
